# Soluble toll like receptor 2 (TLR-2) is increased in saliva of children with dental caries

**DOI:** 10.1186/1472-6831-14-108

**Published:** 2014-08-31

**Authors:** Alyssa Zhao, Corinne Blackburn, Judith Chin, Mythily Srinivasan

**Affiliations:** 1 Department of Oral Pathology, Medicine and Radiology, Indianapolis, IN 46202, USA; 2 Department of Pediatric Dentistry, Indiana University School of Dentistry, Indianapolis, IN 46202, USA

**Keywords:** Saliva, Soluble toll like receptor 2, Dental caries, Biomarker

## Abstract

**Background:**

Dental caries is the most common microbial disease affecting mankind. Caries risk assessment methods, identification of biomarkers and vaccine development strategies are being emphasized to control the incidence of the largely preventable disease. Pattern recognition receptors such as the toll like receptors (TLR) have been implicated as modulators of host-microbial interactions. Soluble TLR-2 and its co-receptor, CD14 identified in saliva can bind the cell wall components of cariogenic bacteria and modulate the disease process. The objective of this study is to determine the potential of salivary sTLR-2 and sCD14 as biomarkers of caries activity and indirect measures of the cariogenic bacterial burden.

**Methods:**

Unstimulated whole saliva was collected from twenty caries free and twenty caries active children between the ages of 5 and 13 years. The concentration of sCD14 and sTLR-2 together with that of the cytokine IL-8 reported to be increased in dental caries was assessed by the enzyme linked immunosorbent assay.

**Results:**

While the level of sCD14 and that of IL-8 was equivocal between the two groups, the sTLR-2 concentration in caries active saliva was significantly higher than that in caries free saliva.

**Conclusions:**

The sTLR-2 in saliva could serve as a potential biomarker for caries activity.

## Background

Dental caries is a multifactorial infectious disease caused by complex interactions between the acid-producing bacteria, fermentable carbohydrates and host factors. Despite being largely preventable, it remains as the most prevalent chronic disease globally [1]. Recent systematic analyses suggest that the incidence of caries exhibits an increasing trend in children aged 2–4 years in the United States [2]. There is evidence for increased prevalence in adults in lower socioeconomic status in European countries. In developing nations the prevalence is increasing in both children and adults [3].

Early detection of white-spot lesions, arresting demineralization and promoting remineralization are some of the preventive clinical methods currently used in dental caries management [4]. In addition considerable emphasis is placed on developing efficient caries risk assessment strategies to determine the likelihood of an individual developing new carious lesion and/or to determine the status of the caries process on individual tooth surfaces [4,5]. As disease of the mineralized tissues of the teeth, pathogenesis of caries involves demineralization of enamel by acid producing bacteria and destruction of the organic substance resulting in cavitation [1,4].

Saliva is recognized as a rich source of host factors capable of modulating the caries process [6,7]. Technological advancements have aided in the characterization of salivary proteomics and peptidomics with the identification of 1444 proteins and 11893 peptides respectively [8]. These protein/peptides belong to different functional classes such as those involved in response to stimulus/stress, antioxidant functions, catalytic functions and enzyme regulators [8,9]. Several salivary components have been assessed for an association with dental caries. While some exhibit weak association, others were equivocal between normal and caries active saliva [9-12]. Assessment of salivary glycoproteins with specific oligosaccharides showed that higher levels of select oligosaccharide that facilitate bacterial colonization at the surface of teeth correlate with caries incidence in young adults [13]. Salivary levels of antimicrobial agents such as alpha defensins, statherin and cystatin S have been suggested as potential risk factors for caries development [10,14].

Toll like receptors (TLR) are germ line encoded receptors that recognize conserved microbial patterns typically shared by large groups of microorganisms. Currently 13 mammalian TLRs and many of their ligands are known [15]. Functioning either alone or in concert with specific co-receptors in recognizing microbial patterns the TLRs act as gate-keepers constantly sampling the environment and eliciting responses to prevent/control infection [15,16]. TLR-2 and TLR-4 have been shown to recognize the peptidoglycan of Gram positive and the lipopolysaccharide of the Gram negative bacteria respectively either alone or in association with the common co-receptor CD14 [17,18]. Odontoblasts localized at the dentino-pulpal surface in healthy teeth have been shown to express TLR-2 and TLR-4. Depending upon the nature of the odontoblastic response caries progression is either suppressed with the formation of reactionary dentin or accelerated leading to pulpal inflammation [19,20]. Microbial invasion of dentin has been shown to upregulate TLR-4 in odontoblasts and mediate TGF-β secretion facilitating collagen synthesis. In addition TLR-4 signaling in odontoblasts also upregulate matrix metalloproteinase-2 promoting cleavage of dentin sialophosphoprotein (DSPP) to dental sialoprotein (DSP) which forms a nucleation site for hydroxyapeptite crystal formation in the newly formed collagen [19]. Stimulation of odontoblast like cells with cell wall components of Gram-positive bacteria elicited cytokine and chemokine secretions in a TLR-2 dependent manner [20]. Elevated levels of cytokines IL-6, TNF-α and IL-8 have been observed in caries active saliva [21].

While primarily membrane associated, recently soluble forms of certain TLRs have been identified in body fluids. It has been suggested that the soluble TLRs function to sequester pathogens [22-25]. Recently, we and others have reported the presence of soluble sCD14 and sTLR-2 in saliva [24,26]. TLR-2 has been shown to recognize the peptidoglycan and the lipotechoic acid of *Streptococcus mutans*, the most common cariogenic bacteria [27]. Considerable evidence suggests a strong correlation between the increased presence of cariogenic bacteria in the plaque biofilm and elevated numbers of the same bacteria in saliva [6,10]. Hence we hypothesized that the level of sTLR-2 and sCD14 in the saliva of caries active individuals will yield an indirect measure of the bacterial burden and act as a biomarker of caries activity. Our data suggest that the sTLR-2 is higher in the unstimulated whole saliva (UWS) of children with active caries lesions.

## Methods

### Study population

In this prospective non-randomized clinical study forty children between 6 and 12 years of age reporting to the pediatric clinics of the Indiana University School of Dentistry were recruited after obtaining informed consent from the patients and guardian. The study was approved by the Institutional Review Board of the Indiana University Purdue University at Indianapolis. Samples were collected only from children with no known oral or systemic disease other than dental caries and no extracted teeth. Oral examinations of children were performed and caries activity recorded. Twenty children (13 boys and 7 girls) were caries free and twenty children (12 boys and 8 girls) were caries active exhibiting four to eight carious lesions requiring restoration.

### Sample collection and processing

All children were instructed to avoid eating and drinking for at least 2 hr prior to saliva collection as described [24,28]. After rinsing the mouth briefly with water, unstimulated whole saliva (UWS) was collected from each child by the passive drooling method for five minutes in pre-chilled tubes. The samples were transported on ice to the laboratory immediately for processing. Each sample was clarified by centrifuging at 4000xg at 4°C for 10 mins and stored in three aliquots at −80°C in CompleteTM Protease Inhibitor Cocktail (Roche, Mannheim, Germany) until further analysis.

### Total protein concentration

The total protein content of pre samples was measured by the Bradford method that involves binding of Commassie blue dye to the proteins [24,25]. The blue protein-dye form was detected at 595 nm using spectrophotometer (Gensys5 spectrophotometer, Thermoelectronic corp, CA). The concentration of the protein in each sample was determined against a standard curve developed using known concentration of bovine serum albumin.

### Enzyme linked immunosorbent assay (ELISA)

For determining sCD14 in saliva a sandwich ELISA kit (R&D Systems, Minneapolis, MN) was used according to manufacturer’s recommendations. Anti-human TLR-2 polyclonal antibody (clone: Imgenex Corporation, San Diego, CA) was used for detection of TLR-2. All UWS samples were depleted of amylase and immunoglobulins by incubating serially with anti-human amylase monoclonal antibody (mAb) (1:2500, catalog #ab8944; Abcam, Cambridge, MA, USA) and protein G beads (Miltenyi Biotec Inc Auburn, CA) at 4°C [24]. The protein content of the precleaned samples was determined as above and precleaned UWS at 1 μg/ml concentration was used for measuring sCD14 and sTLR-2 levels [25]. Preincubation of the UWS with either anti-TLR2 mAb (clone: 1030A5.138, Imgenex Corp, San Diego, CA, USA) (0.5 μg/ml) or with TLR-2 peptides (1 μg/ml) was performed to evaluate the specificity of binding. Bound CD14 and TLR-2 was detected with horse radish peroxidase (HRP) conjugated anti-mouse IgG followed by TMB substrate (Pharmingen, San Diego, CA). Absorbance at 450 nm was read in a microplate reader (model 680: Biorad Laboratories, CA). The concentration of sTLR-2 or sCD14 in pg/ml of salivary proteins was determined using a standard curve of purified recombinant human CD14Fc and TLR-2Fc (R&D Systems) of known concentration. IL-8 concentration in the clarified saliva samples were measured using BD OptEIA3™ ELISA kit.

### Statistical analysis

Differences in the concentration of IL-8, CD14 and TLR-2 between the caries free and caries active groups were determined by Students’ t-test. p value less than 0.05 was considered significant.

## Results and discussion

**Figure 1:**
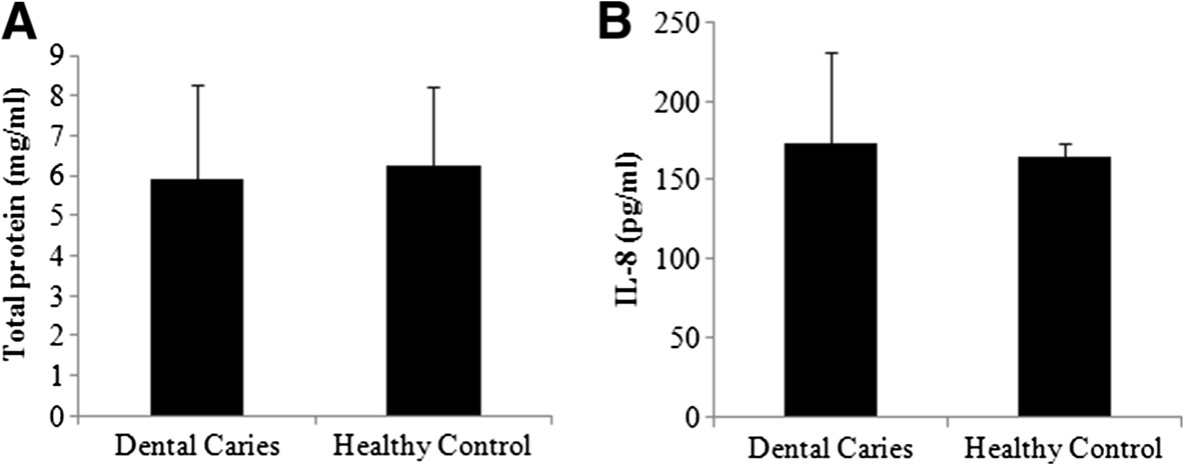
**Total protein concentration in saliva: Clarified whole saliva was assessed for (A) total protein content by spectrophotometry and (B) IL-8 concentration by ELISA.** No significant difference was observed between the caries free and the caries active saliva groups.

Despite advances in early detection methods and efficient preventive measures dental caries remains a highly prevalent chronic disease worldwide. The increasing incidence amongst children in developed countries constitutes a disturbing health concern [2,3]. The demographic details of the study population are given in Table 1. The total protein concentration of human saliva has been shown to exhibit large variations ranging from 0.4 mg/ml to 7.1 mg/ml [29,30]. In our study cohort the total protein concentration measured 6.24+/−1.98 mg/ml in caries free saliva and 5.92+/−2.37 mg/ml in caries active saliva (Figure 1A).

**Table 1:** The demographic data of the study population

**Clinical Data**	**Number recruited**		**Age (yrs)**
	**Male**	**Female**	
Dental caries	12	8	8.76+/−3.42
Healthy control	13	7	7.77+/−2.7

**Figure 2:**
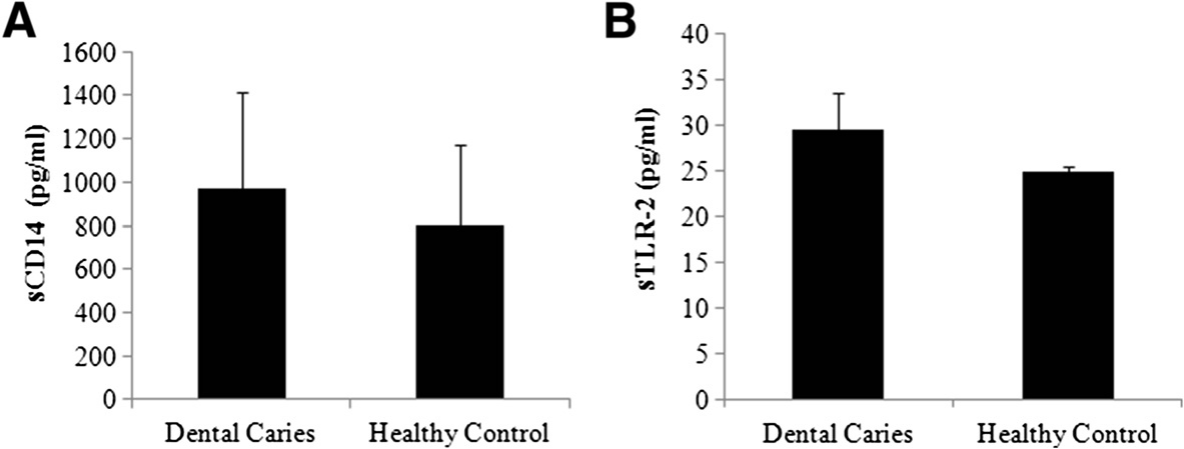
**sCD14 and sTLR-2 concentration in saliva: Clarified saliva was depleted of the high molecular weight abundant proteins, amylase and immunoglobulins, as described in the methods section to increase the sensitivity of detecting of less abundant sCD14 and sTLR-2.** The level of **(A)** sCD14 and **(B)** sTLR-2 was determined by ELISA. * represents p < 0.05.

To the increasing number of salivary proteins are added the soluble forms of pattern recognition receptors, the sTLR-2, sCD14 and sTLR-4 [25,26,31]. The concentration of sTLR-2 in parotid saliva has been reported to be several folds higher than in whole saliva [31]. The source of sTLR-2 could be either active secretion from the gland and/or a result of extracellular cleavage of the membrane bound receptor. We observed that the concentration of sTLR-2 in caries active saliva (29.5+/−3 pg/ml) was significantly higher than that in caries free saliva (24.8+/−0.6 pg/ml) (Figure 2B). Signaling via TLRs in host cells induces cytokine secretion [15]. Previously Gornowicz et al., have reported elevated levels of salivary IL-8 in dental caries [21]. We observed that the salivary IL-8 concentration did not differ significantly between caries free and caries active saliva (Figure 1B). The variable observation between the two studies could be attributed to the differences in the age and the nature of the sample (amylase and Ig depleted vs. undepleted). The concentration of sCD14 in saliva was equivocal in both groups; ranging between 509 pg/ml and 1443 pg/ml in caries free group and between 609 pg/ml and 1829 pg/ml in caries active group (Figure 2A). Previously Bergandi et al., reported complete absence of sCD14 in saliva in children with two to eight carious lesions [28]. The use of pre-cleaned saliva and the method used for measuring sCD14 (Western blot versus sandwich ELISA) could attribute to the observed differences between the Bergandi report and our study.

Based on its strong association with caries incidence multiple studies evaluated salivary levels of *S.mutans* for caries risk prediction with variable results [1,32]. The polymicrobial etiology of dental caries as well as the interaction between salivary proteins and *S.mutans* could contribute to the variability [30,33]. Four major types of salivary protein-microbe interaction have been observed in vitro. These include aggregation, adherence, inhibition/cell-killing, and nutrition [33]. It is postulated that the observed increase in sTLR-2 in caries active saliva may represent a host measure to combat the increased Gram + ve cariogenic bacteria. This suggests that the salivary sTLR-2 level may represent an efficient biomarker for caries activity. The wide variation in the sTLR-2 concentration in caries active saliva could be due to the extent of caries.

## Conclusions

Dental caries is a complex disease, the clinical severity of which depends on the interaction between oral microbes, availability of fermentable carbohydrates and host factors in saliva. This makes identification of predictive risk factors for the carious process very difficult [5,6]. In this report we observed that two soluble proteins, sCD14 and sTLR-2, that act at the microbial host interface are modified in caries active saliva with a later representing a potential biomarker for caries activity. Future studies will correlate the sTLR-2 levels with the cariogenic bacterial counts in saliva.

## Abbreviations

UWS: Unstimulated whole saliva; TLR: Toll like receptor.

## Competing interests

The authors declare that they have no competing interest.

## Authors’ contributions

AZ: Alyssa is a high school sophomore who carried out the ELISA experiments as part of the summer research scholarship program, the Project Seed, co-sponsored by the American Chemical Society and the Indiana Clinical Translations Sciences Institute. She also wrote the first draft of the “Background” section. CB: Corinne is an undergraduate research assistant who helped Alyssa in conducting the ELISA experiments, more so in data analysis. She actively participated in sample processing. She also participated in manuscript writing. JC: Dr. Chin is the pediatric dentist and assisted in the recruitment of study population and sample collection. MS: is the senior investigator overseeing the project including obtaining ethical approvals, experimental design, and data analysis and manuscript preparation. All authors read and approved the final manuscript.

## Pre-publication history

The pre-publication history for this paper can be accessed here:

http://www.biomedcentral.com/1472-6831/14/108/prepub

## References

[B1] Smith DJ Dental caries vaccines: prospects and concerns Crit Rev Oral Biol Med 2002 13 4 335 349 10.1177/154411130201300404 12191960

[B2] Do LG Distribution of caries in children: variations between and within populations J Dent Res 2012 91 6 536 543 10.1177/0022034511434355 22223436

[B3] Costa SM Martins CC Bonfim Mde L Zina LG Paiva SM Pordeus IA Abreu MH A systematic review of socioeconomic indicators and dental caries in adults Int J Environ Res Public Health 2012 9 10 3540 3574 23202762 10.3390/ijerph9103540 PMC3509471

[B4] Anusavice KJ Present and future approaches for the control of caries J Dent Educ 2005 69 5 538 554 15897335

[B5] Hallett KB The application of caries risk assessment in minimum intervention dentistry Aust Dent J 2013 58 Suppl 1 26 34 23721335 10.1111/adj.12047

[B6] Lencova E Broukal Z Spizek J Point-of-care salivary microbial tests for detection of cariogenic species–clinical relevance thereof–review Folia Microbiol 2010 55 6 559 568 10.1007/s12223-010-0090-x 21253899

[B7] Martins C Buczynski AK Maia LC Siqueira WL Castro GF Salivary proteins as a biomarker for dental caries–a systematic review J Dent 2013 41 1 2 8 10.1016/j.jdent.2012.10.015 23142096

[B8] Yan W Apweiler R Balgley BM Boontheung P Bundy JL Cargile BJ Cole S Fang X Gonzalez-Begne M Griffin TJ Hagen F Wolinsky LE Lee CS Malamud D Melvin JE Menon R Mueller M Qiao R Rhodus NL Sevinsky JR States D Stephenson JL Than S Yates JR Yu W Xie H Xie Y Omenn GS Loo JA Wong DT Systematic comparison of the human saliva and plasma proteomes Proteomics Clin Appl 2009 3 1 116 134 10.1002/prca.200800140 19898684 PMC2773554

[B9] Ribeiro TR Dria KJ de Carvalho CB Monteiro AJ Fonteles MC de Moraes CK Fonteles CS Salivary peptide profile and its association with early childhood caries Int J Paediatr Dent 2013 23 3 225 234 10.1111/j.1365-263X.2012.01258.x 22892037

[B10] Guo L Shi W Salivary biomarkers for caries risk assessment J Calif Dent Assoc 2013 41 2 107 109 112–108 23505756 PMC3825179

[B11] Tenovuo J Salivary parameters of relevance for assessing caries activity in individuals and populations Community Dent Oral Epidemiol 1997 25 1 82 86 10.1111/j.1600-0528.1997.tb00903.x 9088696

[B12] Tulunoglu O Demirtas S Tulunoglu I Total antioxidant levels of saliva in children related to caries, age, and gender Int J Paediatr Dent 2006 16 3 186 191 10.1111/j.1365-263X.2006.00733.x 16643540

[B13] Denny PC Denny PA Takashima J Si Y Navazesh M Galligan JM A novel saliva test for caries risk assessment J Calif Dent Assoc 2006 34 4 287 290 292–284 16900986

[B14] Dale BA Tao R Kimball JR Jurevic RJ Oral antimicrobial peptides and biological control of caries BMC Oral Health 2006 6 Suppl 1 S13 10.1186/1472-6831-6-S1-S13 16934114 PMC2147588

[B15] Takeda K Kaisho T Akira S Toll-like receptors Annu Rev Immunol 2003 21 335 376 10.1146/annurev.immunol.21.120601.141126 12524386

[B16] Pulendran B Palucka K Banchereau J Sensing pathogens and tuning immune responses Science 2001 293 5528 253 256 10.1126/science.1062060 11452116

[B17] Kirschning CJ Schumann RR TLR2: cellular sensor for microbial and endogenous molecular patterns Curr Top Microbiol Immunol 2002 270 121 144 12467248 10.1007/978-3-642-59430-4_8

[B18] Moreillon P Majcherczyk PA Proinflammatory activity of cell-wall constituents from gram-positive bacteria Scand J Infect Dis 2003 35 9 632 641 10.1080/00365540310016259 14620147

[B19] Charadram N Farahani RM Harty D Rathsam C Swain MV Hunter N Regulation of reactionary dentin formation by odontoblasts in response to polymicrobial invasion of dentin matrix Bone 2012 50 1 265 275 10.1016/j.bone.2011.10.031 22079283 PMC3246533

[B20] Farges JC Carrouel F Keller JF Baudouin C Msika P Bleicher F Staquet MJ Cytokine production by human odontoblast-like cells upon Toll-like receptor-2 engagement Immunobiology 2011 216 4 513 517 10.1016/j.imbio.2010.08.006 20850890

[B21] Gornowicz A Bielawska A Bielawski K Grabowska SZ Wojcicka A Zalewska M Maciorkowska E Pro-inflammatory cytokines in saliva of adolescents with dental caries disease Ann Agric Environ Med 2012 19 4 711 716 23311795

[B22] Iwami KI Matsuguchi T Masuda A Kikuchi T Musikacharoen T Yoshikai Y Cutting edge: naturally occurring soluble form of mouse Toll-like receptor 4 inhibits lipopolysaccharide signaling J Immunol 2000 165 12 6682 6686 10.4049/jimmunol.165.12.6682 11120784

[B23] LeBouder E Rey-Nores JE Rushmere NK Grigorov M Lawn SD Affolter M Griffin GE Ferrara P Schiffrin EJ Morgan BP Labeta MO Soluble forms of Toll-like receptor (TLR)2 capable of modulating TLR2 signaling are present in human plasma and breast milk J Immunol 2003 171 12 6680 6689 10.4049/jimmunol.171.12.6680 14662871

[B24] Srinivasan M Kodumudi KN Zunt SL Soluble CD14 and toll-like receptor-2 are potential salivary biomarkers for oral lichen planus and burning mouth syndrome Clin Immunol 2008 126 1 31 37 10.1016/j.clim.2007.08.014 17916440

[B25] Zunt SL Burton LV Goldblatt LI Dobbins EE Srinivasan M Soluble forms of Toll-like receptor 4 are present in human saliva and modulate tumour necrosis factor-alpha secretion by macrophage-like cells Clin Exp Immunol 2009 156 2 285 293 10.1111/j.1365-2249.2009.03854.x 19292767 PMC2759477

[B26] Isaza-Guzman DM Aristizabal-Cardona D Martinez-Pabon MC Velasquez-Echeverri H Tobon-Arroyave SI Estimation of sCD14 levels in saliva obtained from patients with various periodontal conditions Oral Dis 2008 14 5 450 456 10.1111/j.1601-0825.2007.01400.x 18938271

[B27] Hong SW Baik JE Kang SS Yun CH Seo DG Han SH Lipoteichoic acid of Streptococcus mutans interacts with Toll-like receptor 2 through the lipid moiety for induction of inflammatory mediators in murine macrophages Mol Immunol 2014 57 2 284 291 10.1016/j.molimm.2013.10.004 24216318

[B28] Bergandi L Defabianis P Re F Preti G Aldieri E Garetto S Bosia A Ghigo D Absence of soluble CD14 in saliva of young patients with dental caries Eur J Oral Sci 2007 115 2 93 96 10.1111/j.1600-0722.2007.00437.x 17451497

[B29] Chiappin S Antonelli G Gatti R De Palo EF Saliva specimen: a new laboratory tool for diagnostic and basic investigation Clin Chim Acta 2007 383 1–2 30 40 17512510 10.1016/j.cca.2007.04.011

[B30] Rudney JD Does variability in salivary protein concentrations influence oral microbial ecology and oral health? Crit Rev Oral Biol Med 1995 6 4 343 367 10.1177/10454411950060040501 8664423

[B31] Kuroishi T Tanaka Y Sakai A Sugawara Y Komine K Sugawara S Human parotid saliva contains soluble toll-like receptor (TLR) 2 and modulates TLR2-mediated interleukin-8 production by monocytic cells Mol Immunol 2007 44 8 1969 1976 10.1016/j.molimm.2006.09.028 17081611

[B32] Pitts N Duckworth RM Marsh P Mutti B Parnell C Zero D Post-brushing rinsing for the control of dental caries: exploration of the available evidence to establish what advice we should give our patients Br Dent J 2012 212 7 315 320 10.1038/sj.bdj.2012.260 22498529

[B33] Scannapieco FA Saliva-bacterium interactions in oral microbial ecology Crit Rev Oral Biol Med 1994 5 3–4 203 248 7703323 10.1177/10454411940050030201

